# Supplementary material for: Plasticity Comparison of Two Stem Cell Sources with Different Hox Gene Expression Profiles in Response to Cobalt Chloride Treatment during Chondrogenic Differentiation
Source: Biology (Basel). 2024 Jul 24;13(8):560. doi: 10.3390/biology13080560 (PMC11352031; doi:10.3390/biology13080560)
Supplement: Supplementary file 1 [file biology-13-00560-s001.zip › Table S1.pdf]

Table S1: RT-PCR primers

Gene	Sequence of the selected primer pair	Length of amplicon (nucleotide)
Hox A5	Forward:5´-CCCTTCAATCTCGTTTAGT-3´<br>Reverse: 5´- TCTCATCAAGTCACCTCTA -3´	171
Hox A7	Forward:5´- ACAACAAATCACAGGTCAAA -3´<br>Reverse: 5´-AGCAAAGGAGCAAGAAGTC-3´	119
Hox C10	Forward:5´- CGAAGCGAAAGAGGAGATA-3´<br>Reverse:5´- AGCGTCTGGTGTTTAGTAT-3´	102
β actin	Forward: 5´-AATCGTGCGTGACATTAAG-3´<br>Reverse: 5´-GAAGGAAGGCTGGAAGAG-3´	178
