# Supplementary material for: Plasticity Comparison of Two Stem Cell Sources with Different Hox Gene Expression Profiles in Response to Cobalt Chloride Treatment during Chondrogenic Differentiation
Source: Biology (Basel). 2024 Jul 24;13(8):560. doi: 10.3390/biology13080560 (PMC11352031; doi:10.3390/biology13080560)
Supplement: Supplementary file 1 [file biology-13-00560-s001.zip › Table S2.pdf]

**Table S2:** qPCR program

| Step | Time | Temperature |
| --- | --- | --- |
| Initial denaturation | 30 seconds | 95 ºC |
| PCR | | |
| | 5 seconds | 95 ºC |
| | 34 seconds | 60 ºC |
| | 15 seconds | 95 ºC |
| Repeat cycles | Repeat cycle steps 40 times | |
